# A wearable light-touch contact device for human balance support

**DOI:** 10.1038/s41598-021-85687-4

**Published:** 2021-04-01

**Authors:** Keisuke Shima, Koji Shimatani, Mami Sakata

**Affiliations:** 1 Faculty of Engineering, Yokohama National University, Yokohama, 240-8501 Japan; 2 Prefectural University of Hiroshima, Mihara, 723-0053 Japan

**Keywords:** Biomedical engineering, Preventive medicine, Sensory processing

## Abstract

There is an urgent need for the development of ways to address the major issue of falls among today’s globally aging population. The authors here outline a new approach referred to as virtual light-touch contact to mitigate postural sway during ambulatory and stationary periods, and propose a wearable light-touch (WLT) system featuring a virtual partition around the user that allows determination of virtual forces resulting from related contact. The data produced are used to create vibrotactile fingertip feedback, which supports comprehensive perception of the partition. Non-impaired subjects were recruited to support evaluation of the prototype system (incorporating tactile stimulation and motion-capture technology), with outcomes showing successful mitigation of postural sway in a heel-to-toe tandem stance. Research performed with 150 able-bodied volunteers to validate the performance of the new set-up (incorporating an acceleration sensor and a voice coil motor to render the light-touch effect) suggested that the proposed WLT approach supports human balance on a level comparable to that of the light-touch effect.

## Introduction

Motor function disorders such as Parkinson’s disease are associated with accidents involving falls due to the increased postural sway they cause. In this context, there are concerns that the incidence of such accidents in Japan may increase in the future due to the characteristics of the nation’s super-aging society. In 2007, the related locomotive syndrome was identified by the Japanese Orthopaedic Association as a mobility issue related to compromised locomotive capacity. In order to reduce the number of fall accidents resulting from impaired postural control among elderly people^1–3^, there is a need to develop a support method for postural control in daily life.

Canes, walking frames and other aids^4,5^ help the older population with ambulatory and balance-related instabilities, taking weight off the legs and creating a larger support base^3^. Although assistive devices designed to enhance postural control are considered very effective, some research outcomes have suggested that their misuse or usage on stairs and in certain other environments can exacerbate motor function disorders or actually increase the risk of falling^3^.

Fingertip contact cues are known to provide stimuli that support postural sway reduction despite being insufficient to actually stabilize the subject physically^6^. Extensive research has been conducted on this technique, known as light-touch contact (LT)^6–10^. Riley et al.^7^ reported on work involving standing subjects with eyes closed and LT using a curtain, with a conclusion indicating reduced postural sway. In a similar field, Shimatani et al. reported on LT with subjects touching a piece of hanging paper at various heights^9^ and illuminance levels^8^ among aged people. In the same vein, Dickstein et al. also reported LT’s effectiveness in reducing ambulatory postural instability^10^. Meanwhile, influences from LT with noise applied to the fingertips were reported by Magalhaes et al.^11^. Despite the successes reported in these studies, LT cannot be applied without the presence of a curtain or another actual partition in the real world. With this in mind, there is a need for research on the application of LT with no actual partition toward the establishment of a technique to mitigate stationary and ambulatory postural instability. Such work can be expected to help address the issue of falls among the senior population.

Here, the authors outline the concept of a wearable device to enhance stationary and ambulatory stability using LT without the need for a curtain or another actual partition. The approach is based on motion-capture technology using acceleration sensors and IR cameras in combination with vibrotactile stimulation, and incorporates the placement of a virtual partition around the user along with determination of virtual forces resulting from user contact. The information collected is used to create vibrotactile fingertip feedback to the user via a compact lightweight stimulator to mitigate instability using somatosensory input. Previous experiments conducted by the authors on virtual light touch indicated its potential for use in the mitigation of instability^12–14^. The validity of the concept is discussed here based on experimental evaluation of a wearable light-touch device with over 150 volunteers in the 1920s–1960s age bracket.

## Results

**Figure 1:**
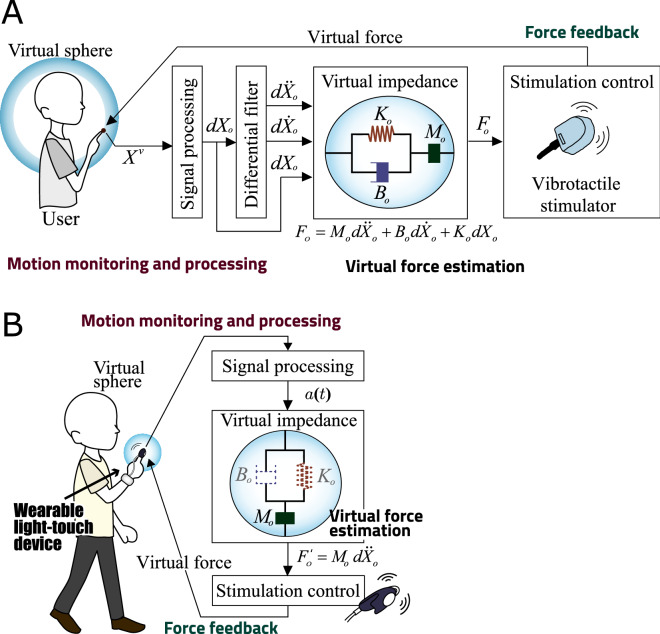
Overview of the proposed virtual light-touch system.The system involves the steps of motion monitoring and processing, virtual force estimation based on non-contact impedance, and force feedback provision to the user via a vibrotactile stimulator based on motion. (**A**) Basic system involving 3D motion capture and non-contact impedance with full parameters. (**B**) Wearable light-touch device with a simple acceleration sensor and a vibrotactile stimulator based on the simplest equation of motion (acceleration only).

Figure 1 gives an overview of the proposed wearable light-touch contact system, whose operation involves the three steps of motion monitoring/processing, virtual force estimation and force feedback provision via vibrotactile stimulation. Figure 1A outlines basic virtual provision of the light-touch effect, and Fig. 1B highlights the advanced wearable system for virtual light-touch implementation. Wearable light touch is a novel method that can be adopted to support postural control without the use of a physical partition. Its application is as follows:

1. A virtual partition is set around the user.
2. Contact with the virtual partition is used to estimate virtual force.
3. Estimation of virtual force is premised on sensations received via the fingertips.

In the system, a virtual partition is generated around the user, and data on virtual forces determined from the non-contact impedance^15^ parameters registered when the partition is set are fed back to the user via fingertip vibrotactile stimulation.

The advanced wearable LT system (Fig. 1B) consists of a three-axis acceleration sensor, a small tactile stimulator and a microcomputer with a battery. Fingertip acceleration data are processed by the microcomputer and the vibrator is controlled in line with the virtual reaction forces calculated.

**Figure 2:**
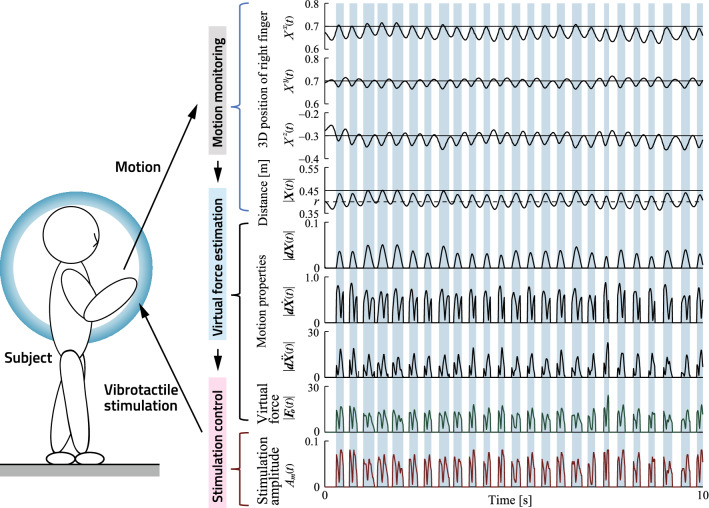
Experimental results for VLT conditions. Top to bottom: 3D finger position, norms of trunk-fingertip distance, motion properties (norm of distance into the partition, velocity and acceleration), estimated virtual force, system-generated stimulation amplitude. Usage is also shown in Movie S1.

**Figure 3:**
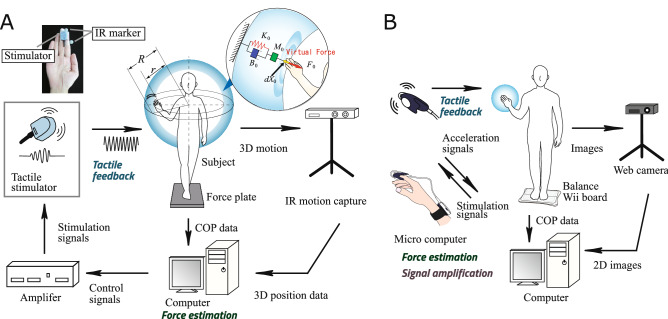
Hardware components and experimental equipment for the prototype system. (**A**) 3D motion capture-based light-touch system for experiments 1 and 2. IR marker attached to the subject’s trunk and fingertip with virtual force computation based on subject motion. The computer also generates stimulation signals for fingertip feedback. (**B**) Wearable light-touch device used for experiment 3. The subject’s motions are monitored using the fingertip acceleration sensor. Virtual forces are determined on a microcomputer based on acceleration, and fingerpad feedback is provided in line with motion. A webcam is used to record subject movement.

Using the proposed device, the user can generally sense the virtual force caused by contact with the partition in the air, which helps to reduce postural sway in a standing state. Examples of measured data for VLT tasks are given in Fig. 2, which shows (from top to bottom) the 3D position of the right finger *X*(*t*), the norm of finger positions |*X*(*t*)|, the distance norm $|{{dX_o}}(t)|$ from the subject’s trunk, the velocity norm $|{{\dot{dX_o}}}(t)|$, the acceleration norm $|{{\ddot{dX_o}}}(t)|$, the virtual force norm $|{{F_o}}(t)|$ and the stimulation signal amplitudes $A_m(t)$. The shaded areas indicate time intervals between instances of contact with the virtual partition ($r \le |{{X}}(t)| < R$; shown in Fig. 3A). Here, the subject experiences fingertip somatosensory input via vibrotactile stimulation in line with motion properties. VLT system testing is shown in Movie S1.

Validity was examined in three standing-posture experiments involving six healthy volunteers using the motion capture system (Fig. 3A), and the effectiveness of the non-contact impedance parameters for the area around the user was investigated. The validity of the wearable device was also examined via postural sway experiments involving 156 volunteers using a simple evaluation system (Fig. 3B).

### Volunteers

The details of all 156 volunteers recruited for the experiments are shown in Table 1. Grasping strength and functional reach test scores were recorded for most of the volunteers, who also completed a questionnaire asking: (1) Do you have any chronic conditions? (2) How many times a week do you exercise? (3) Have you fallen in the last year? The results indicated that none of the volunteers had any impairment and none had fallen within the past year. The table also summarizes exercise habits and test score acquisition ratios.

**Table 1:** Subject information used in experiments.

Condition	Age	Average ages (years)	Sex		Exercise habits/week (%)			Grip strength (kg)		FRT ($10^{-3}$m)	Ac. rates (%)
			M	B	3 or more	1 or 2	No or less	Right	Left		
Exp. 1 and 2	–	$23.17 \pm 1.33$	9	0	–			–	–	–	–
Exp. 3	1920s	$24.07 \pm 2.62$	20	10	6.67	33.33	60.00	$38.71 \pm 9.71$	$35.67 \pm 7.87$	$37.57 \pm 6.88$	90.00
	1930s	$34.63 \pm 2.63$	12	18	13.33	33.33	53.33	$40.81 \pm 9.90$	$38.25 \pm 9.86$	$38.07 \pm 6.11$	70.00
	1940s	$43.73 \pm 2.99$	6	24	13.33	23.33	63.33	$30.40 \pm 8.56$	$27.66 \pm 7.65$	$36.81 \pm 6.94$	86.67
	1950s	$54.43 \pm 2.67$	11	19	13.33	20.00	66.67	$32.73 \pm 8.84$	$31.05 \pm 6.76$	$36.15 \pm 6.12$	90.00
	1960s	$64.90 \pm 3.14$	4	26	26.67	40.00	33.33	$27.60 \pm 7.31$	$26.61 \pm 6.50$	$35.63 \pm 6.36$	86.67

Test scores and exercise habits were not examined for the subjects in experiments 1 and 2.

*Ac. rates* acquisition rates of test score, *FRT* functional reach test score.

### Mitigation effect of virtual light touch (experiment 1)

**Figure 4:**
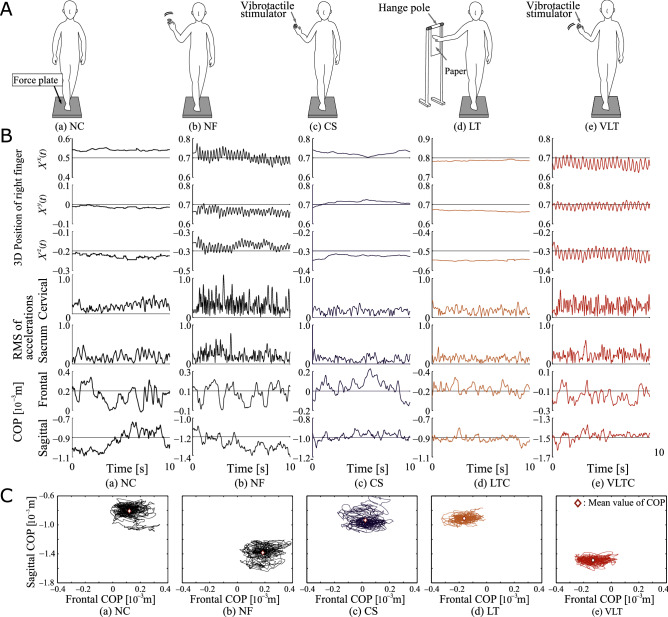
Tasks and results from experiments 1 and 2. The figures show (a) the subject’s posture with no contact (NC) anywhere, (b) NC with the hand moving to touch the virtual partition without feedback (NF), (c) NC with constant stimulation applied to the fingertips (CS), (d) light touch with a piece of paper hanging to the subject’s right (LT), and (e) virtual light touch with the right hand (proposed method; VLT). (**A**) Tasks for each condition (**B**) Experimental results; from top to bottom: 3D fingertip position; acceleration RMS; (**C**) COP trajectories for each task.

Experiments were conducted to evaluate the validity of the light-touch effect and the proposed light-touch system using vibrotactile stimulation with six healthy volunteers. Each volunteer was asked to retain a tandem (heel-to-toe) stance on the force plate with the eyes closed and perform five tasks as follows: (a) no contact (NC) anywhere, (b) no contact with the hand moving to touch the virtual partition without feedback (NF), (c) no contact with constant fingertip stimulation (CS), (d) light-touch contact (LT) with a piece of paper (length: 297 cm; width: 210 cm) hanging to the subject’s right side, and (e) virtual light touch (VLT) with the right hand (proposed method). Figure 4A shows task scenes from the experiment.

Examples of the experimental results for 10 s are given in Fig. 4B, which shows (from top to bottom) the 3D position of the right finger *X*(*t*), root mean squares of calculated acceleration signals and the subject’s center of pressure (COP). Figure 4C shows COP track areas as determined from the force plate over a period of 1960s for each condition. Movie S2 shows scenes from the experiments.

**Figure 5:**
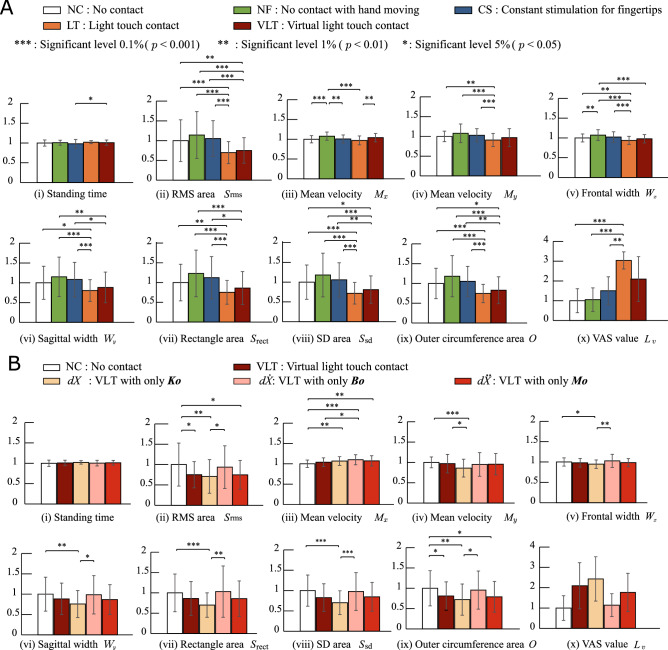
Experimental results for six subjects for each evaluation index (experiments 1 and 2). (**A**) Mitigation effect of virtual light touch in a tandem stance (experiment 1) (**B**) Examination of relationships between non-contact impedance parameters and human standing (experiment 2).

Figure 5A shows the evaluation results for the tasks with each index. The results of a multiple comparison test using the Holm method for each index showed no significant differences in the standing time *T* (Fig. 5A(i)) for each task (without CS condition). The averages of the root mean square area of COP $S_{\text{rms}}$ in LT and VLT was significantly lower at the 0.1% and 1% levels for NC. The averages of the mean velocity $M_y$ for LT was significantly lower than that for NC. The frontal width $W_x$ and sagittal width $W_y$ represent the width of COP sway. The values for LT are significantly lower than that for NC. The rectangular area $S_{\text{rect}}$ for LT were significantly lower that for NC. The area of SD $S_{\text{sd}}$ and the outer circumference area *O* for VLT were significantly smaller for NC. The averages of the visual analogue scale $L_v$ (sensation of stability as expressed by the subjects themselves) indicated by evaluation for all subjects were $1.00 \pm 0.60$ (NC), $1.05 \pm 0.60$ (NF), $1.51 \pm 0.70$ (CS), $3.34 \pm 0.43$ (LT) and 2.10 ±1.13 (VLT).

### Analysis of impedance parameters (experiment 2)

To evaluate the effectiveness of somatosensory input via vibrotactile stimulation, experiments were conducted in a standing state with varying impedance parameters on six volunteers with 3D motion capture (Fig. 3A). The volunteers were asked to retain a tandem stance with eyes closed on a force plate and to perform three tasks involving (1) NC, (2) LT and (3) VLT with the right hand. The basic VLT impedance parameters for estimation of virtual forces were evaluated with:

1. All parameters (stiffness, viscosity and inertia)
2. Stiffness parameter only (other parameters set to 0)
3. Viscosity parameter only (other parameters set to 0)
4. Inertia parameter only (other parameters set to 0)

Ten trials were conducted for each condition with a task duration exceeding 60 s.

Figure 5B presents the comparison results when using VLT and when performing NC, which include 10 indices. The VLT results showed lower index values from subjects’ COP than those of NC. Significant differences were observed between the conditions of NC and the four tasks for some indices. In particular, the results show that $S_{\text{rms}}$ and *O* indices are lower when the subjects used VLT compared to when they performed NC. Since postural sway is greater in the frontal direction than sagittal direction during the tandem standing posture, VLT affects largely balance of a person in the former direction than the latter. That was confirmed by the decrease of the postural sway size and range indicated by $S_{\text{rms}}$ and *O*, respectively. No significant difference was observed between the conditions of VLT with all parameters (wine red bars) and VLT with inertia parameter only (red bars). These outcomes demonstrate that the effects of VLT can be provided using only single parameters such as acceleration ${\ddot{dX_0}}(t)$. As acceleration signals can be measured using a simple acceleration sensor, VLT can be realized using a compact, lightweight device.

### Evaluation of the wearable light-touch device (experiment 3)

**Figure 6:**
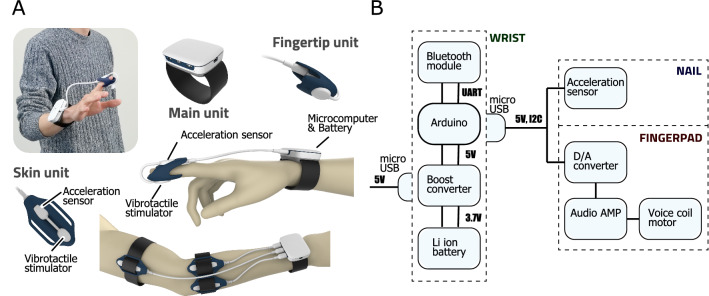
Proposed wearable light touch (WLT) device. (**A**) Image and hardware composition of the WLT device in experiment 3. The main unit with a microcomputer and Li ion battery can be worn on the wrist. The acceleration sensor and vibrotactile stimulator are attached to the fingernail and fingerpad, respectively, and the device supports fingertip mode and body mode for acceleration monitoring and provision of stimulation. (**B**) Device circuit components. The device can be charged via USB and communicate with the computer via Bluetooth. The stimulation signals generated are sent to the voice coil motor via a D/A converter and amplifier.

A prototype VLT system with an acceleration sensor was developed from the results of the above experiments. This simple sensor is a compact, lightweight model popularly used in recent years, and lends itself to the light-touch effect for a new compact and wearable device. Here, the virtual impedance parameters of the proposed set-up can be modified as a simple mass model (Fig. 1B). Figure 6 illustrates the proposed wearable light-touch device (WLT), which consists of a three-axis acceleration sensor (attached to the top of the subject’s fingernail) and a voice coil motor (attached to the fingerpad side) covered with dark blue silicon rubber. Figure 6A shows the image and hardware composition of the WLT device, and Fig. 6B shows the device circuit components. The voice coil motor provides somatosensory input to the subject’s fingerpad via vibrotactile stimuli based on estimated virtual forces. The sensor and motor are connected to a microcomputer on the subject’s wrist that processes acceleration signal data to determine the relevant virtual forces and generate control signals for the vibrotactile stimulator. The device also has three other ports for identical sensors and stimulators that can be attached to other parts of the body for similar data harvesting. Movie S3 shows scenes from the experiments in tandem and closed-leg stances with eyes closed using the proposed WLT.

In the wearable VLT system, virtual forces are estimated based on a simple equation of motion when the acceleration norm exceeds the threshold $\alpha _{\text{th}}$. Subjects experience somatosensory input in line with their motion via vibrotactile stimulation. Experiments were performed to evaluate the validity of the proposed device with 150 healthy volunteers in the 1920s–1960s age bracket (30 individuals in each age 10-year group) based on the experimental setup shown in Fig. 3B. COP was determined from a force plate (Balance Wii Board, Nintendo Inc.) and motion was captured using a simple web camera as in the experiments. The volunteers were asked to retain a closed-leg standing posture with eyes closed, and to perform two tasks based on (1) no contact anywhere with the hand moving to touch the virtual partition without feedback (NF) and (2) VLT using the acceleration-based wearable device (WVLT).

**Figure 7:**
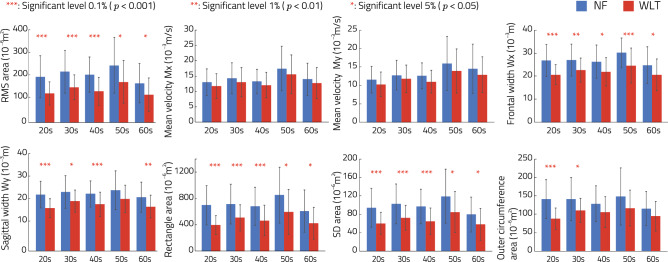
Experimental results for 150 subjects with WLT (experiment 3). NF (blue bars) represents results from the condition of no contact anywhere with the hand moving to touch the virtual partition without feedback in a closed-leg stance. WLT (red bars) represents results from a closed-leg stance with the WLT device. Each graph shows COP indices for RMS area, mean velocity for the frontal and sagittal sides, frontal and sagittal width, rectangle area, SD area and outer circumference area. ***, ** and * represent significant differences of 0.1, 5 and 1%, respectively.

Figure 7 shows the average evaluation indices observed during each task. All indices were lower than those for NF at all ages, and significant differences were observed between NF and WLT in the RMS area of COP, the frontal width, the SD area and the rectangle area. Significant differences were seen in and between NC and WVLT at the 0.1, 1 or 5% levels in each index. This demonstrates that postural sway can be mitigated by WVLT.

## Discussion

During VLT-task contact periods, vibrotactile stimulation was applied to feed back virtual force norms $|{{F_o}}(t)|$ based on the finger motion properties ($|{{dX_o}}(t)|$, $|{{\dot{dX_o}}}(t)|$, $|{{\ddot{dX_o}}}(t)|$) as shown in Fig. 2. Movie S1 shows how the subjects experienced this feedback and controlled the tandem standing posture using the prototype system.

Figure 4 and Movie S2 show that the finger position waves for NF and VLT changed periodically with hand movement, and that the waves of the root mean square calculated from acceleration signals varied greatly as compared to those of the other three tasks (NC, CS, LT). The movie indicates how the subject’s body sway was mitigated by LT and VLT in comparison to NC, NF and CS conditions. Large changes in the waves for the surface evaluation index values $M_x$ (the mean velocity) and $W_x$ (the frontal width) between NC and NF are also observed (Fig. 5A). The changes are thought to have been influenced by waving hand motions. However, $W_x$ for VLT was significantly lower than those for NF at the 0.1% level. Thus, it can be inferred that VLT suppresses increased acceleration caused by waving hand motions. In addition, there was no significant increment between NC and VLT for other indices. These results indicate that waving hand motion with VLT increases acceleration rather than body sway. This assumption is also supported by the fact that the area of COP tracks for VLT as measured from the force plate was much smaller than for NC, NF and CS, and as small as that for LT (Fig. 4C).

The experimental results indicate that postural instability in a tandem stance with the eyes closed is mitigated by LT. In addition, VLT notably reduces values of the root mean square are of COP $S_{\text{rms}}$, the standard deviation (SD) area $S_{\text{sd}}$, and the outer circumference area of COP *O*. $S_{\text{rms}}$, $S_{\text{sd}}$ and *O* for VLT were significantly lower than for NC at the 1% and 5% levels, and a similar tendency (no significant differences) were seen in $S_{\text{rms}}$, $S_{\text{sd}}$ and *O* for LT. This is presumed to result from tactile feedback based on virtual forces estimated from contact with the virtual partition because postural sway was not mitigated in two tasks (no contact with the hand moving to touch the virtual partition without feedback and application of constant stimulation to the subjects’ fingertips). Postural sway was also reduced when the subject sensed 3D coordinates in the space of the sphere. Additionally, as there was no significant difference in the standing time *T* for each task (without CS condition), it was presumed that there was no large postural sway exceeding the dimensions of the support base during the experiments. However, some subjects were unable to retain a tandem stance for 60 s, and it can be seen that VLT conditions slightly extended the standing time for such subjects. VAS values also suggested the effectiveness of the balance aid for standing with the proposed VLT.

The efficacy of the VLT method in producing effects similar to those of the LT approach for reducing instability is suggested by the above outcomes. In VLT, the terms $M_o$, $B_o$ and $K_o$ represent the virtual inertia, viscosity and stiffness matrices associated with the virtual partition. When the subject touches the partition, virtual force is fed back via vibrotactile stimulation. Here, the VLT effect may be influenced by the impedance parameters $M_o$, $B_o$ and $K_o$. Experiment 2 examined how the standing state changes with virtual partition parameters. The experiment results demonstrate that the effects of VLT can be provided using only single parameters such as acceleration, velocity and distance, and show that simple vibrotactile stimulation based on the norm of acceleration signals can be used for virtual force feedback to mitigate body sway with the light-touch effect. Here, the basic VLT system requires the 3D motion capture system for trunk/fingertip monitoring to allow estimation of virtual forces using a full set of impedance parameters. However, the use of such a large and complex motion capture system is impractical in daily life. The acceleration-based VLT system can be applied as a balance aid with a simple, lightweight and compact sensor and motor, and has the potential for widespread adoption among elderly people.

Based on these results, experiment 3 was conducted to evaluate the effectiveness of the proposed wearable light-touch (WLT) device with 150 volunteers in comparison with the no-contact condition. The volunteers were asked to touch the virtual partition without feedback (NF). Figure 7 shows that most indices for the WLT condition were significantly lower than those for the NF condition, indicating that the LT effect created by the proposed device supports balance among elderly people with issues retaining a closed-leg stance. The baseline of each index in the 1960s age bracket was lower than that for subjects in their 1950s, which is attributed to the length of exercise habits among the former in comparison to other age brackets. These results indicate that the proposed simple stimulation device significantly mitigated body sway in all age groups (1920s–1960s) via the light-touch effect. From Movie S3, it can be seen that the WLT device supports balance in closed-leg and tandem stances.

## Conclusion

The authors here outline an approach referred to as virtual light-touch contact (VLT) that helps to increase the physical stability of the user with no need for an actual partition. The technique allows the effects of light-touch contact (LT) to be created via the application of tactile stimulation depending on virtual forces generated through contact with a virtual partition set around a user based on a non-contact impedance model^15^.

In the experiments, postural sway observed during five tasks involving VLT and LT was monitored using a motion capture system, acceleration sensors and a force plate, and the results obtained were compared. It was found that VLT helps to reduce the postural sway of subjects in a tandem stance without the use of the physical partitions required for LT. It was also observed that postural sway was not mitigated in a tandem stance with the conditions of two of the tasks (no contact with the hand moving to touch the virtual partition without feedback, and application of constant stimulation to the subjects’ fingertips). These results verify that VLT is suitable for human postural stabilization using tactile feedback based on virtual forces estimated from contact with a virtual partition.

The second set of experiments showed that simple acceleration signals from hand movements can be used to produce the light-touch effect virtually. The results also showed that simple linear vibrotactile stimulation based on amplitudes of acceleration supports human balance. Accordingly, the proposed combination of a compact, lightweight acceleration sensor and a vibrator of the type found in smartphones and various other devices can produce the virtual light-touch effect using the proposed method.

The results of comparison between COPs for NF and WLT (experiment 3) show that the light-touch effect can be realized with a simple wearable device developed for users in all the age groups examined here. The device is practical for use in daily life, and supports postural stability in a wide range of environments. In previous work, Shimatani et al. researched LT effects with subjects touching a piece of paper hung at various heights^9^, with results indicating reproduction of the effect at all heights. Research conducted by Ruth et al. also highlighted the potential importance of LT’s role in supporting ambulatory stability^10^. As the proposed device can be used to perceive virtual forces at any height and location in the air, the user’s posture is aided by wearing the device while standing and walking based on the light-touch effect, thereby the device might have a potential to reduce the risk of falls. Thus, the proposed device enables realization of the light-touch effect, giving it a potentially significant impact in today’s super-aging societies.

### Related work, limitations and future work

The proposed device allows realization of the LT effect in human usage. Here, let us consider the effects of the proposed device based on consideration of the LT effect. LT helps to mitigate postural sway when the subject lightly touches surrounding objects with the fingertips, as reported by Jeka et al.^6^. As the contact forces involved are less than 1 N according to several studies, LT has been proposed as a viable tactile feedback-based approach involving active touching to reduce postural sway^6,16^. The results of a study conducted by Kouzaki et al.^17^ indicated that LT was ineffective in supporting postural stability when a compression block was applied to the upper arm to numb the hand.

A variety of studies on the effects of LT have been reported with focus on the subject’s state (e.g., eye status (closed or open) and touching height and attention to subject’s fingertips), the types of fixed objects involved, and the touching method used. Initial investigation of postural stabilization with subjects assuming different stances clarified that LT influenced postural control in patients with bilateral vestibular loss or blindness^6,16,18^. Further studies focused on effects relating to age differences and the presence of certain disorders. Tremblay et al.^19^ and Baccini et al.^20^ compared the effects of LT in old people and young people, while Chen et al.^21^ examined its influence on postural control in infants. Experiments on LT in patients with peripheral neuropathy and in children with developmental coordination disorders have also been reported^22,23^. Light levels in user environments also influence effectiveness^8^. Ishigaki et al.^24^ examined the effects of LT in relation the subject’s relevant fingertip, with results showing that brain activity in the left primary sensorimotor cortex area and the left posterior parietal cortex area reflect sensorimotor information processing and sensory integration for the LT effect. The sensorimotor cortex area was found to be most closely related to the LT effect.

In the late 1990s, Jeka et al. reported that the effects of LT were not influenced by fixed-surface friction^25^. Results from research by Riley et al. also showed that improved stability was achieved from contact with a curtain around the subject, while also indicating that a rigid contact surface was unnecessary^7^. In 2008, Nagano et al. reported a slight reduction of body sway in relation to subjects touching their own upper legs^26^. It is also known that lightly touching objects such as a piece of hanging paper^9^ or the elbow of another person^27^ supports postural control.

A number of studies have addressed approaches to LT. Clapp et al.^28^ determined its effects with subjects in a normal bipedal stance, while Rabin^29^ investigated postural stabilization in relation to subjects touching a fixed surface with the right upper limb restricted. The results of an experiment involving the provision of light and passive tactile cues to the leg via a ground-fixed device also indicated that passive tactile stimuli can help to reduce postural sway^30,31^.

These related works indicate that the effects of postural stabilization via LT are unaffected by the type of fixture the subject touches and that only minimal finger contact force is required. As a variety of studies have shown that the user benefits from the effects of LT involving the sensation of vibrotactile stimulation via the fingertips from contact with a fixed object, it can be reasonably assumed that the technique contributes to the reduction of postural sway. However, the LT effect did not support balance well for subjects with impaired attention ability and/or sensory impairments (e.g., visual, somatosensory and vestibular issues for standing posture control). Although the effect aided balance slightly, posture could not be recovered once a fall had started (meaning that the approach does not provide comprehensive protection against falls), and the technique was ineffective in relation to significant instability caused by various other conditions (e.g., internal conditions among subjects and standing on unstable/uneven ground). The proposed wearable device produces effects similar to those of LT, enabling slight improvement of postural control via somatosensory input.

In future research, the authors plan to discuss optimal parameters for the non-contact impedance model and stimulation methods for enhanced mitigation of body sway. In recent years, various exoskeleton-based devices have been proposed to support balance in standing and ambulatory situations^32,33^. As the device described here works synergistically with these devices, the proposed method has wide potential for application in daily life. A further aim is to consider its applicability in support for various situations such as walking, climbing stairs and vehicle boarding/alighting.

## Methods

### System architecture

Figure 1 shows an overview of the prototype system, which consists of a 3D motion sensor (OptiTrack V120: Trio, Spice Inc.) to determine the user’s trunk and finger positions, a vibrotactile stimulator (VBW32C25, Audiological Engineering Corp; length: 1 inch; width: 0.73 inches; thickness: 0.42 inches; weight: 6.5 g) to feed tactile stimulation signals back to the user, an amplifier for stimulation, and a computer for estimation of virtual forces based on contact with the virtual partition and the generation of control signals for the stimulator. In the system, a virtual partition is first set around the user’s trunk based on measurements taken using the motion sensor, and the computer then determines the distance between the fingers and the partition. When the fingers come into contact with the partition, the computer generates control signals for the stimulator based on virtual forces estimated from the user’s motions. These signals are then amplified and sent to the tactile stimulator attached to the user’s fingertips.

The advanced wearable light-touch device is shown in Fig. 1B. The system consists of a simple voice coil motor, a three-axis acceleration sensor and a microcomputer. A virtual sphere is set around the user’s fingertip, and the microcomputer generates control signals to stimulate the fingerpad in line with the user’s finger/hand motions. Further details are provided below for a basic system (Fig. 1A) and an advanced wearable system (Fig. 1B).

### Motion monitoring and preprocessing

Finger and/or trunk positions/motions need to be precisely established for virtual partition setting. In the basic system, this is achieved using infrared markers attached to two fingertips and a 3D motion sensor (sampling frequency: $f_s$ Hz) to determine the 3D position vector *X*(*t*) $\in \mathfrak {R}^3$ based on the marker origins at the trunk center. The fingertip position vector *X*(*t*) is applied to determine acceleration ${\ddot{X}}$(*t*) and fingertip velocity ${\dot{X}}$(*t*) via the use of differentiation filters, and the Euclidian norm of *X*(*t*)(|*X*(*t*)|) is determined.

For the wearable system (Fig. 1B), position information for the subject’s fingertip was acquired using three-dimensional acceleration signals. The value *a*(*t*) $\in \mathfrak {R}^3$ for the fingertip was monitored with an acceleration sensor, and the Euclidean norm |*a*(*t*)| was calculated to produce exercise information relating to the subject.

### Estimation of virtual forces

This approach involves the monitoring of virtual forces resulting from contact with a virtual partition around the subject’s trunk using a non-contact impedance model^15^. Figure 3A highlights this approach, which incorporates the setting of a virtual hollow sphere (internal radius: *r*; external radius: *R*) from the trunk center point. Figure 3A outlines the determination of virtual non-contact impedance between the center of the subject’s trunk and the sphere, with $M_o$, $B_o$ and $K_o$ respectively expressing virtual inertia, viscosity and stiffness matrices relating to the sphere. The normal vector from the inside of the sphere to a fingertip piercing the virtual partition toward the outside is expressed by

1 $$\begin{aligned} {dX_o}(t) = {X}(t) - r{n}(t) , \end{aligned}$$

where the vector *n*(*t*) $\in \mathfrak {R}^3$ is defined as

2 $$\begin{aligned} {n}(t) = \frac{{X}(t)}{|{X}(t)|}. \end{aligned}$$

In consideration of the vector ${dX_o}(t)$ and non-contact impedance, the virtual force ${{F_o}}(t)$ exerted from the virtual partition to a fingertip inside the sphere (*r* $\le$ |*X*(*t*)| < *R*) is expressed as

3 $$\begin{aligned} {F_o}(t)= -(M_o{\ddot{dX_o}}(t)+B_o{\dot{dX_o}} (t) +K_o{dX_o}(t)) \end{aligned}$$

here, differentiation filters are used to determine acceleration ${\ddot{dX_o}}(t)$ and velocity ${\dot{dX_o}} (t)$ based on the displacement vectors ${dX_o}(t)$. For a fingertip outside the sphere, ${F_o}(t)$ has a zero value. The non-contact impedance model can thus be applied to determine virtual forces from sphere contact.

The applicability of the simplest non-contact impedance condition based on inertia/acceleration was examined in experiment 2 to support virtual force estimation. The proposed wearable system (Fig. 1B) involves the setting of a threshold $a_{\text{th}}$ for acceleration ${\ddot{dX_o}}(t)$ based on acceleration sensor data. Virtual forces are determined using Eqs. (4) and (5) when the acceleration norm passes this level.

4 $$\begin{aligned} {F_o}(t)&= - M_o {\ddot{dX_o}}(t) \end{aligned}$$

5 $$\begin{aligned} {\ddot{dX_o}}(t)&\cong \left\{ \begin{array}{cl} {|{a}(t)| - a_{\text th} } &{} (|{a}(t)| > a_{\text th}) \\ 0 &{} (|{a}(t)| \le a_{\text {th}}) \end{array} \right. \end{aligned}$$

### Force feedback using vibrotactile stimulation

A tactile feedback unit containing a compact wearable actuator is used to apply virtual forces stemming from partition contact. As the precision of such actuators for this purpose is limited, the authors considered in particular the LT characteristic of requiring forces as low as $\le 1$ N toward the development of an approximate technique involving the use of a compact vibrotactile stimulator to apply virtual forces. These stimulators can be used to apply skin-surface vibration at amplitudes up to $A_{\text{max}}$ (frequency: $f_z$ Hz). In this context, numerous studies have been conducted on the propensity for sensation of vibrotactile stimulation on human skin. In this case, the norm of the virtual force vector ${F_o}(t)$ is referenced to calculate signal amplitudes $A_m(t)$:

6 $$\begin{aligned} A_m(t)=\left\{ \begin{array}{ll} k|{F_o}(t)|  (k|{F_o}(t)| < A_{\text{max}}) \\ A_{\text{max}}  (k|{F_o}(t)| \ge A_{\text{max}}) \\ \end{array},\right. \end{aligned}$$

here, *k* represents the constant gain. When $k|{F_o}(t)|$ exceeds the maximum amplitude, $A_m(t)$ is expressed as $A_m(t) = A_{\text{max}}$.

The proposed approach involves the use of a D/A converter for the digitization of computer-generated sinusoidal signals (as per Eq. (6)). A low-power audio amplifier is then used to process data for fingertip application via the vibrotactile stimulator to enable sensation of virtual forces resulting from virtual-partition contact.

### Experiment conditions

This research was performed in accordance with relevant guidelines and regulations and approved by the ethics committee of the Faculty of Health and Welfare at Prefectural University of Hiroshima (14MH009). Written informed consent was received from all subjects, who also attended face-to-face sessions to learn about the experiments in advance. Informed consent to publish this manuscript and the subject’s information/images was also obtained from subjects.

Experiments 1 and 2 were conducted to verify the effectiveness of LT and the proposed VLT system with six healthy male volunteers (average age: $23.2 \pm 1.33$). Two triaxial accelerometers (MA3-04Ac, Microstone Co., Ltd.) and a force plate (TF-4060, Tec Gihan Co., Ltd) were used to measure postural sway. The location of the vibrotactile stimulator and the attached infrared markers is given Fig. 3A. The stimulator was attached to the tip of the subject’s index finger, and the infrared markers were attached to the lateral side of the index and middle fingers for position measurement.

The non-contact impedance parameters were set as $M_o = 1.0$ kg, $B_o = 10.0$ Ns/m, $K_o = 50.0$ N/m, $A_{\text{max}} = 0.08$ V and $k = 0.005$ V/m by trial and error. The parameters *r* and *R* to be set for the virtual partition were adjusted, the internal diameter *r* was set to the optimal position for touching as indicated by the subject, and the external diameter was set at a distance between the trunk and the finger position with the arm fully extended. The location of the paper used in LT was adjusted to the optimal position for touching as indicated by the subject. The sampling frequency of the 3D motion capture system and the acceleration sensor was 100 Hz, and that of the force plate was 5 kHz. The stimulation frequency $f_z$ was 250 Hz. The number of trials was 11, and the subjects were asked to retain a tandem stance as much as possible for a period exceeding 60 s.

In experiment 3, the wearable VLT device was evaluated with 150 volunteers in the 1920s to 1960s age bracket. COP was monitored using a Balance Wii Board from Nintendo Inc., and a standard web camera was used to film the subjects’ motions (evaluation set-up: Fig. 3B; force plate/acceleration sensor frequency: 100 Hz). The subjects were asked to retain a closed-leg stance with eyes closed for 20 s or more in each task in a single trial; some subjects in the 1960s age bracket were unable to retain a tandem stance.

The following indices were calculated to evaluate postural stability. All indices were used for experiments 1 and 2, and 2–9 were used for experiment 3.

1. Standing time *T*
2. Root mean square (RMS) of acceleration of the seventh cervical vertebra $R_s$
3. Mean velocity of COP $M_x$
4. Mean velocity of COP $M_y$
5. Frontal width of COP $W_x$
6. Sagittal width of COP $W_y$
7. Rectangle area of COP $S_w$
8. Standard deviation (SD) area of COP $S_{\text{sd}}$
9. Outer circumference area of COP *O*
10. Visual analogue scale of standing stability $L_v$

The standing time *T* was defined as the period during which the tandem stance was maintained, and was calculated from the number of data *N* measured by the force plate (sampling frequency: $f_p$ Hz).

7 $$\begin{aligned} T=\frac{N}{f_p} \end{aligned}$$

Indices (2)–(9) are commonly utilized to evaluate body sway, and are calculated using the COP ($x_{n}$, $y_{n}$). Here, $x_{n}$ and $y_{n}$ represent the frontal and sagittal COP, respectively. The root mean square area of the COP $S_{\text{rms}}$ and the mean velocities $M_x$ and $M_y$ are defined as the dispersion from the average center of pressure and the average velocity of postural sway, respectively

8 $$\begin{aligned} M_{r}= & {} {\frac{\pi }{N}\sum _{n=1}^{N}\{(x_{n} - {\overline{X}})^2 + (y_{n} - {\overline{Y}})^2\}} \end{aligned}$$

9 $$\begin{aligned} M_{v}= & {} {\frac{1}{T}}\sum _{n=1}^{N}\sqrt{{e_{n}^{x}}^2 + {e_{n}^{y}}^2} \end{aligned}$$

here, the average and variation of the COP on the *x*- and *y*-axes are set as ${\overline{X}}$, ${\overline{Y}}$ and $e_{n}^{{x}^2}$, $e_{n}^{{y}^2}$, respectively.

The frontal width $W_x$ and sagittal width $W_y$ are defined as the difference between the maximum and minimum value of each COP coordinate as calculated using Eqs. (10) and (11)

10 $$\begin{aligned} W_{x}= & {} \left| \mathop {\text{arg~max}}\limits _{1 \le n \le N} x_{n} - \mathop {\text{arg~min}}\limits _{1 \le n \le N} x_{n}\right| \end{aligned}$$

11 $$\begin{aligned} W_{y}= & {} \left| \mathop {\text{arg~max}}\limits _{1 \le n \le N} y_{n} - \mathop {\text{arg~min}}\limits _{1 \le n \le N} y_{n}\right| \end{aligned}$$

A rectangular area $S_{\text{rect}}$ indicates the movement range of the COP calculated using Eqs. (12) and (13), and the SD area $S_{\text{sd}}$ is calculated using standard deviation

12 $$\begin{aligned} S_{\text{rect}}= & {} W_{x}W_{y} \end{aligned}$$

13 $$\begin{aligned} S_{\text{sd}}= & {} \pi \sigma _{x}\sigma _{y} \end{aligned}$$

here, the standard deviations of the COP on the *x*- and *y*-axes are set as $\sigma _{x}$ and $\sigma _{y}$.

To determine the outer circumference area of COP *O*, the frontal width of COP is divided into 1 mm sections, and the minimum and maximum points of the sagittal COP for each section point are calculated. The resulting vertex coordinates are then arranged in a clockwise manner and defined as the periphery point of COP $P_i=(p_i^x, p_i^y)$. The outer circumference area of COP *O* is calculated as

14 $$\begin{aligned} O= \frac{1}{2} \left| \sum _{i=1}^n (p_i^x p_{i+1}^y - p_{i+1}^x p_i^y)\right| \end{aligned}$$

when $i=n$, it is defined that $n+1=1$.

In addition, a visual analogue scale (VAS) was used to evaluate the subjects just after the experiment. VAS evaluation ranges from ‘Not at all stable’ on the far left to ‘Very stable’ on the far right. The subjects were asked to evaluate their level of postural stability by marking a position on the line during each task. The VAS value $L_v$ is the distance between the far left and the point marked by the subject.

All indices computed using data recorded from all subjects, and significant differences between tasks were evaluated using the Holm method.

## Supplementary Information

Supplementary Information.

Supplementary Video 1.

Supplementary Video 2.

Supplementary Video 3.

## Data Availability

All data needed to evaluate the conclusions in the paper are present in the paper or the Supplementary Materials. Contact K. Shima. (shima@ynu.ac.jp) for additional data and other materials.
