# Supplementary material for: A wearable light-touch contact device for human balance support
Source: Sci Rep. 2021 Apr 1;11:7324. doi: 10.1038/s41598-021-85687-4 (PMC8016895; doi:10.1038/s41598-021-85687-4)
Supplement: Supplementary file 1 — Supplementary Information. [file 41598_2021_85687_MOESM1_ESM.pdf]

# Supplementary Materials for

## A Wearable Light-touch Contact Device for Human Balance Support

Keisuke Shima, Koji Shimatani, Mami Sakata

Correspondence to: shima@ynu.ac.jp

**This PDF file includes:**

Fig. S1

**Other Supplementary Materials for this manuscript include the following:**

Movies S1 to S3

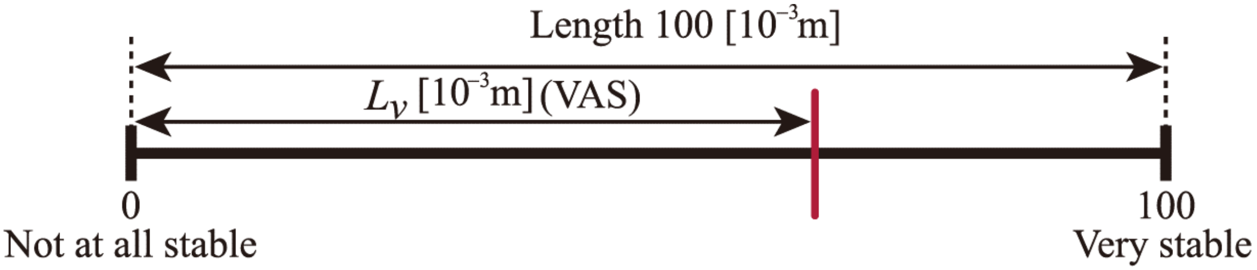


**Fig. S1.**
Visual analog scale (VAS) for experiment 1 and 2.

**Movie S1.**

VLT standing. The movie shows how subjects experienced this feedback and controlled upright posture in tandem using the prototype system.

**Movie S2.**

Comparison of conditions in a tandem stance with eyes closed. The movie indicates how the subject's body sway was mitigated by LT and VLT via somatosensory finger input in comparison to NC, NF and CS conditions.

**Movie S3.**

Comparison of conditions for NF and WLT in closed-leg and tandem stances
Fig. S2. Visual analog scale (VAS) for experiment 1 and 2.
